# A Systematic Scoping Review of Access to Safe Drinking Water in Sub-Saharan Africa: Mapping Literature on Determinants, Interventions, and Policy Implications over the Past Decade and the Path Forward

**DOI:** 10.4314/ejhs.v34i5.10

**Published:** 2024-09

**Authors:** Emmanuel Udochukwu Osisiogu, Kehinde Peter Akinrotoye, Amanda Eyram Banini, Raphael Eyram Amemo

**Affiliations:** 1 Department of Science Laboratory Technology, Dr. Hilla Limann Technical University, Wa, Ghana; 2 Department of Microbiology, College of Biosciences, Federal University of Agriculture, Abeokuta, Ogun State, Nigeria; 3 Department of Biomedical Sciences, University of Health and Allied Sciences, Ho, Ghana

**Keywords:** Water access, Sub-Saharan Africa, Determinants, Interventions

## Abstract

**Background:**

Access to safe drinking water remains a critical challenge in Sub-Saharan Africa, driven by a complex mix of environmental, political, social, economic, and infrastructural factors. This scoping review aims to map the literature on water access in Sub-Saharan Africa over the past decade.

**Methods:**

We conducted a comprehensive search of academic databases and grey literature from January 2013 to the present. We included peer-reviewed quantitative, qualitative, and mixed-methods research, as well as reviews and reports focusing on factors influencing water access and related interventions in Sub-Saharan Africa. Data were extracted on study characteristics, key determinants, proposed solutions, and outcomes.

**Results:**

A total of 137 studies were included. Commonly reported determinants included droughts, climate change, conflict, governance, gender, wealth, education, poverty, and inadequate infrastructure. Identified potential interventions included infrastructure development, water quality monitoring, climate adaptation, governance reforms, decentralized management, targeted subsidies, and integrated water resources management. However, most studies described barriers rather than evaluating solutions.

**Conclusions:**

Persistent inequities in water access are driven by interconnected factors such as poverty, governance, gender, and infrastructure. Implementing integrated solutions is crucial, with a shift from problem identification to evaluating contextualized interventions across sectors. Dedicated implementation research is needed to translate knowledge into action, advancing water security and achieving Sustainable Development Goal 6 in the region.

## Introduction

Globally, access to safe water is essential for human health, socio-economic development, and poverty reduction (1-3). However, significant inequities persist, with Sub-Saharan Africa facing particularly severe challenges. Only 24% of the population in Sub-Saharan Africa has access to safe and readily available water within their premises, compared to 94% in Europe and North America (4). This lack of improved water access disproportionately affects the poor and marginalized groups, exacerbating health and economic disparities (5,6).

Waterborne diseases such as diarrhoea, cholera, typhoid, polio, and hepatitis A, place a substantial burden on areas with limited access to safe water (7). It is estimated that 88% of global diarrhoeal deaths occur in children under five in Sub-Saharan Africa and South Asia (8). Furthermore, inadequate access to clean water impacts education, livelihoods, and poverty reduction efforts (6,9-12), with women often spending significant time collecting water, which limits their opportunities for socioeconomic development and advancement (13).

Access to potable water in Sub-Saharan Africa is restricted by a complex interplay of political, socio-economic, environmental, and infrastructural factors (6,14,15). Even in water-rich regions, insufficient infrastructure for water harvesting, treatment, and distribution limits access for many communities (16). Rapid urbanization strains existing, often outdated, water supply systems (17-21), while seasonal droughts negatively affect water availability (19,20). Persistent access gaps arise from urbanization, inefficient water delivery systems, governance constraints, and weather-related risks.

Privatization of water resources and resultant price increases deepen socio-economic divides in water access (20-24). Vulnerable groups, including women, children, the poor, disabled, refugees, and rural dwellers, are disproportionately affected (25, 26). These intersectional issues highlight the need to view water access through an equity and social justice lens, rather than solely as an infrastructural issue (27).

Improving access to safe water in Sub-Saharan Africa is both a critical public health priority and a moral imperative, justifying its inclusion in the Sustainable Development Goals (28). However, our understanding of the complex factors affecting clean water availability remains incomplete. A broad scoping review can help map the extent of research activity, identify knowledge gaps, and inform policy and future research directions (29).

On World Water Day, it is highlighted that over 1.42 billion people, including 450 million children, primarily in Sub-Saharan Africa, live in areas with high or extremely high water vulnerability (25). This equates to 1 in 5 children globally lacking sufficient water for daily needs. This scoping review seeks to systematically map the literature on access to safe water in Sub-Saharan Africa published over the past decade, synthesizing current knowledge on determinants, interventions, and policy implications to recommend evidence-based solutions for improving water access in the region.

## Methods

This scoping review adhered to the methodological frameworks proposed by Arksey and O'Malley (30) and the Joanna Briggs Institute (31).

**Eligibility criteria:** Studies were selected based on the following criteria.

**Population**: Individuals residing in sub-Saharan Africa

**Concept**: Access to safe drinking water sources

**Context**: Factors affecting access to safe water and interventions/policies aimed at improving water availability

**Types of sources**: Peer-reviewed primary studies (both quantitative and qualitative), reviews published in academic journals, and grey literature from governmental bodies, NGOs, and policy institutes.

**Period**: January 2013 to present

**Language**: English

**Geographical scope**: Countries in sub-Saharan Africa

**Information sources**: The search strategy covered key databases including PubMed (for Medline), Embase, CINAHL, Web of Science, Scopus, and Engineering Village, using relevant search terms. Grey literature was sourced through targeted searches of websites such as WHO, UNICEF, USAID, World Bank, and African government databases.

**Search strategy**: A search strategy was developed for PubMed (for Medline), combining keywords related to water, access, Africa, and health, along with database-specific index terms. The search strategy was peer-reviewed by a librarian following the PRESS checklist to ensure comprehensiveness and accuracy (32).

**Figure 1:**
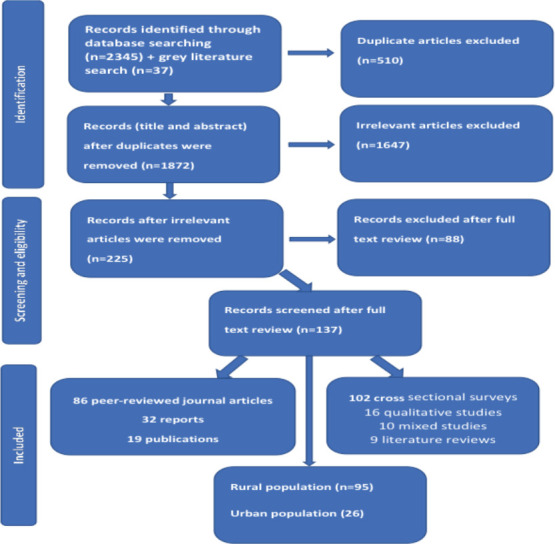
Literature search and study inclusion

**Screening and selection**: All identified records were imported into EndNote, and duplicates were removed. Title/abstract and full-text screenings were conducted independently by two reviewers according to the eligibility criteria, with disagreements resolved through discussion. The PRISMA flowchart illustrates the screening process and exclusions at each stage (Figure 1).

**Data extraction**: A data extraction form was designed to collect pertinent information from the included studies, covering study details (authors, year, and location).

**Study design and methods**: Key themes/findings related to water access determinants, interventions, and policies were used. The draft form was piloted on a subset of studies and refined based on feedback. Data extraction was performed independently by two reviewers, with consensus reached on final versions.

**Data synthesis**: An iterative narrative synthesis approach was employed, involving tabulation, thematic analysis, and textual descriptions. Extracted information was categorized to map evidence availability and identify gaps. Determinants of water access were organized according to the WHO framework, covering environmental, political, social, economic, and infrastructural domains (4). Interventions and policies were mapped and synthesized, with descriptive statistics used to summarize study designs, locations, populations, and other key features.

## Results

The database searches yielded 2,345 records, with an additional 37 records from grey literature sources. Following PRISMA guidelines for searching, screening, and including papers, a total of 137 studies were included in this review.

**Determinants of safe water access**: The key determinants of safe water access identified in the studies correspond closely with the WHO framework, categorized as follows:

**Environmental**: Water scarcity due to droughts or seasonal rainfall variations was a major barrier (38 studies). Studies indicated reduced access to improved water sources during dry seasons compared to rainy seasons (33, 34). Climate change was also noted to worsen water stress, with projections of declining groundwater recharge rates and deteriorating water quality if mitigation measures are not implemented (35).

**Political**: Political factors included conflicts and instability (24 studies), governance issues such as corruption (31 studies), and insufficient governmental prioritization of water access (44 studies). Armed conflicts often disrupt water infrastructure, impacting access (36). Wealthier countries with robust institutions manage water scarcity better, even if poorer nations have more water resources (37).

**Social**: Social determinants included gender, socioeconomic status, and education levels. Studies found that women and girls face greater water access challenges due to cultural norms assigning water collection responsibilities to them (17 studies). Higher wealth and education levels were associated with better water access (29 studies). Marginalized groups, such as refugees and nomadic populations, also experience water access gaps (38).

**Economic**: Key economic determinants were poverty (43 studies) and water affordability (38 studies). Individuals in lower wealth quintiles had reduced access to safe water compared to those in higher quintiles (39). Financial barriers, such as user fees for public standpipes or yard taps, limited access for impoverished households, especially in urban informal settlements (40). Unemployment and limited livelihood opportunities were linked to the use of unimproved water sources (13).

**Infrastructural**: Inadequate infrastructure, including piped water systems, boreholes, protected wells, and rainwater catchment systems, was a significant barrier (62 studies). Rural areas were particularly affected due to insufficient infrastructure and maintenance (18, 37). Rapid urbanization strained existing, often aging, pipe networks and treatment facilities (17). Distance and time required to reach water sources were linked to the use of unsafe water (49 studies).

### Interventions and Policies to Improve Water Access

The review identified several interventions and policies aimed at improving water access:

**Infrastructure Expansion**: New piped networks, wells, and rainwater harvesting, particularly in rural and impoverished urban areas (38 studies).

**Water Quality Monitoring**: Implementing water safety planning and monitoring (24 studies).

**Watershed Management**: Climate change adaptation strategies such as artificial recharge and desalination (15 studies).

**Governance reforms**: Addressing corruption and increasing budgets (20 studies)

**Community-based management**: Decentralized models (32 studies)

**Targeted subsidies and tariff models**: Promoting affordability and cost recovery (18 studies)

**Gender transformative approaches**: Addressing the burdens of water collection (13 studies)

**Integrated management**: Combining WASH and water resources management (14 studies)

However, 23 studies noted that interventions often fail to achieve desired outcomes due to the limited adoption of comprehensive approaches addressing the intersecting environmental, political, social, economic, and infrastructural dimensions.

## Discussion

This scoping review analyzed 137 studies on safe drinking water access in sub-Saharan Africa published over the past decade. The evidence reveals that multiple, complex, and interconnected factors—spanning environmental, political, social, economic, and infrastructural domains—contribute to persistent water access gaps in the region. Rather than a single dominant factor, it is the interplay of these elements that limits access and affects vulnerable populations.

The studies highlighted issues such as poverty, governance weaknesses, gender inequities, and inadequate infrastructure, aligning with global initiatives like the Sustainable Development Goals. Progress towards universal access to safe drinking water has been slow, with only 24% of sub-Saharan Africans having basic water services available on-premises (4).

Potential solutions identified include infrastructure expansion, governance reforms, decentralized management, targeted subsidies, gender-transformative approaches, climate adaptation, and integrated water resources management. Yet, many studies focus on barriers rather than actionable interventions, and integrated approaches remain scarce despite increasing calls for their adoption.

Realizing equitable water security in sub-Saharan Africa requires not only technical understanding of the determinants but also evidence on how to implement comprehensive, cross-sectoral actions. The Human Right to Water and Sanitation framework offers a basis for holistic implementation and monitoring. Achieving water security involves not only addressing technical challenges but also empowering marginalized groups and overcoming political and economic constraints.

This review underscores the need for action research to translate knowledge into practical solutions, focusing on overcoming interdisciplinary and systemic barriers. The goal is to advance from understanding the challenges to implementing and evaluating solutions that match the scale and complexity of the water crisis. Only then can the human right to water in Africa move from rhetoric to reality, contributing to the achievement of Sustainable Development Goal 6.
